# Pharmacokinetics of Salmeterol and Fluticasone Propionate Delivered in Combination via Easyhaler and Diskus Dry Powder Inhalers in Healthy Subjects

**DOI:** 10.1089/jamp.2017.1437

**Published:** 2018-09-26

**Authors:** Merja Kirjavainen, Leena Mattila, Mikko Vahteristo, Jani Korhonen, Satu Lähelmä

**Affiliations:** Orion Pharma, Orion Corporation, Espoo, Finland.

**Keywords:** bioequivalence, Easyhaler, fluticasone propionate, lung deposition, salmeterol

## Abstract

***Background:*** Easyhaler^®^ dry powder inhaler (DPI) containing salmeterol and fluticasone propionate was developed for the treatment of asthma and chronic obstructive pulmonary disease. Three different Salmeterol/fluticasone Easyhaler test products (Orion Pharma, Finland) were compared against the reference product Seretide^®^ Diskus^®^ DPI (GlaxoSmithKline, United Kingdom) to study whether any of the test products are bioequivalent with the reference.

***Methods:*** Open and randomized pharmacokinetic four-period crossover study on 65 healthy volunteers was performed in a single center to compare the lung deposition and total systemic exposure of salmeterol and fluticasone propionate after administration of single doses (two inhalations of 50/500 μg/inhalation strength) in fasting conditions. Blood samples were drawn before dosing and at frequent time points between 2 minutes and 34 hours after dosing for determination of drug concentrations. The primary variables for total systemic exposure and lung deposition of fluticasone propionate were maximum concentration of the concentration–time curve (C_max_) and area under the concentration–time curve from time zero to the last sample with quantifiable concentration (AUC_t_). For salmeterol, the primary variables for total systemic exposure were C_max_ and AUC_t_ and for lung deposition C_max_ and AUC up to 30 minutes after study treatment administration (AUC_30min_).

***Results:*** One of the Easyhaler test products met all the criteria for bioequivalence with the reference. The 96.7% confidence intervals (CIs) for the test/reference ratios of fluticasone propionate C_max_ and AUC_t_ were 0.9901–1.1336 and 0.9448–1.0542, respectively. Ninety percent CIs for salmeterol C_max_, AUC_30min_, and AUC_t_ ratios were 1.0567–1.2012, 1.0989–1.2255, and 1.0769–1.1829, respectively. Median salmeterol time to maximum concentration (t_max_) was 4.0 minutes. Median fluticasone propionate t_max_ was from 1.5 to 2.0 hours. Terminal elimination half-life was 11 hours for salmeterol and 9–10 hours for fluticasone propionate.

***Conclusions:*** Salmeterol/fluticasone Easyhaler was shown to be bioequivalent with the reference product.

## Introduction

Salmeterol is a potent and selective β_2_-adrenoceptor agonist that, in addition to the regular management of asthma, is approved in various countries for the maintenance therapy of chronic obstructive pulmonary disease (COPD). The bronchodilatory effect of salmeterol lasts for more than 12 hours. As the effect begins slowly and reaches its maximum within 2–3 hours after a single dose, it is not suitable for the treatment of acute asthma attack.^(1,2)^

Fluticasone propionate is a synthetic, trifluorinated corticosteroid with mainly glucocorticoid activity. It is a highly lipophilic molecule that binds avidly to lung tissue. When inhaled, fluticasone propionate has a dose-dependent anti-inflammatory action in the airways, resulting in reduced symptoms and fewer asthma exacerbations.^(3,4)^

Combination of salmeterol and fluticasone propionate is widely used in asthma and COPD management. Concurrent use of salmeterol and fluticasone propionate does not result in any untoward interaction that would affect the pharmacodynamic or pharmacokinetic profiles of the individual drugs, or their adverse effect profiles.^(5)^

Easyhaler^®^ is a dry powder inhaler (DPI) developed by Orion Corporation Orion Pharma (Espoo, Finland). It is currently marketed for the administration of salbutamol, beclometasone, budesonide, formoterol, and budesonide/formoterol combination.

Orion Pharma has now developed a combination formulation of salmeterol and fluticasone propionate for the Easyhaler inhaler (hereafter Salmeterol/fluticasone Easyhaler). In the development, the European guideline on the requirements for clinical documentation for orally inhaled products (OIPs) has been followed.^(6)^ It describes how to demonstrate therapeutic equivalence between an inhaled second entry product and an originator product. Based on the required *in vitro* comparisons, it is known that Salmeterol/fluticasone Easyhaler differs from the reference medicinal product Seretide^®^ Diskus/Accuhaler^®^ (hereafter Seretide Diskus) with respect to some characteristics, and therefore, pharmacokinetic studies are needed.

The objective of the study presented here was to demonstrate noninferiority in total systemic exposure and bioequivalence (BE) in lung deposition between at least one Salmeterol/fluticasone Easyhaler test product and Seretide Diskus.

## Materials and Methods

### Study treatments

Seretide Diskus 50/500 μg/inhalation, inhalation powder (GlaxoSmithKline, United Kingdom) was used as the reference product. Three product variants, Easyhaler test products A, B, and C, of Salmeterol/fluticasone Easyhaler 50/500 μg/inhalation, inhalation powder (Orion Corporation, Orion Pharma, Espoo, Finland) were used as test products.

The Easyhaler products differed with respect to powder formulation, the main difference being the particle size distribution of lactose carrier. The delivered doses (DDs) of the test products matched those of the reference product. For test products A, B, and C and Seretide Diskus, the mean DDs (standard deviation) for salmeterol were 44 (1.8), 47 (3.2), 47 (3.1), and 45 (1.9) μg/dose, and the mean DDs for fluticasone propionate were 457 (17.0), 497 (42.5), 486 (28.2), and 474 (19.3) μg/dose. In addition, the inhaler of test product B had a modified mouthpiece to enhance powder dispersion. The air flow resistance of product B inhaler was slightly lower than the resistance of products A and C inhaler, being 0.036 and 0.044 √kPa·min/L, respectively.

The product batches tested were selected to be representative of the typical test and reference product performance. Reference product fine particle doses (FPDs, the mass of particles under 5 μm) were studied according to the *in vitro* testing of DPIs established by the European Pharmacopoeia monograph Preparations for Inhalation^(7)^ using Next Generation Impactor (apparatus E). The FPDs of the tested reference product batch were close to the mean FPDs of 35 reference product batches. The Easyhaler batches studied were found to be representative among the manufactured production scale batches.

### Study subjects

Healthy male and female subjects aged 18–60 years old with body mass index 19–30 kg/m^2^, weight at least 50 kg, good general health, a forced expiratory volume in 1 second at least 80% of the predicted normal value, and who gave written informed consent (IC) were enrolled. Smokers were excluded as well as pregnant or breastfeeding females and those of childbearing potential not using adequate contraception. Verbal and written information about the study was given to the study subject candidates before recruitment. Adequate time and opportunity were given to inquire about details of the study and to decide whether or not to participate before signing the IC.

### Study design

This was an open, randomized, single dose pharmacokinetic study performed in a single center. The bioanalytical laboratory was blinded in this study. The subjects were randomized to receive single doses of three test products and a reference product according to a four-period, four-treatment crossover design. The study consisted of a screening period, 4 treatment days, and an end-of-study visit. The treatment days were separated by washout periods of at least 7 days. Before each study treatment administration, the subjects stayed overnight at the study center.

The study (EudraCT 2016-000714-29) was conducted according to the principles of the Declaration of Helsinki of the World Medical Association and in compliance with the protocol, good clinical practice (GCP) as detailed in ICH/135/95, and the applicable regulatory requirements. The study protocol, subject information and IC form, and subject diaries were reviewed and approved by the National Committee on Medical Research Ethics (TUKIJA), Finland (approval number 149/13/03/00/2016), and the national regulatory authority of Finland (FIMEA) (approval number 78/2016) before start of the study.

### Assessments

During the treatment periods, a single dose of two inhalations of Salmeterol/fluticasone Easyhaler or Seretide Diskus was inhaled in the morning after an overnight fast in the study center (Clinical Pharmacology Unit, Orion Pharma). The time interval between the inhalations was 45 seconds. The study treatments were inhaled according to Seretide Diskus patient information leaflet (PIL)^(8)^ and the planned Easyhaler PIL (similar administration as with the other Easyhaler products on the market). A standard lunch was served 4 hours after dosing.

As correct study treatment administration was essential in this study, training of the inhalation technique was carried out during the screening and before each study treatment administration. In addition, separately for all the products, the target peak inspiratory flow (PIF) rates were defined based on the median PIF values recorded in a study on asthmatic and COPD patients.^(9)^ If the administration was not successful for some reason, the study period could be terminated before further pharmacokinetic sampling and rescheduled for another day.

Peripheral venous blood samples were drawn for the determination of salmeterol and fluticasone propionate concentrations in plasma at the following time points: before study treatment administration (0 hours) and at 2 minutes, 4 minutes, 6 minutes, 15 minutes, 30 minutes, 45 minutes, 1 hour, 1 hour 30 minutes, 2 hours, 2 hour 30 minutes, 3 hours, 4 hours, 7 hours, 12 hours, 24 hours, and 34 hours after study treatment administration.

Salmeterol and fluticasone propionate concentrations in plasma were determined by a validated achiral ultra performance liquid chromatography-tandem mass spectrometry method (PPD, Inc., Middleton, WI).^(10)^ The lower limit of quantification for both analytes was 1.00 pg/mL. The bioanalytical analyses were performed according to the principles of applicable good laboratory practice and GCP. The bioanalytical laboratory was kept blinded and analyses of the samples were conducted without information on treatment.

The pharmacokinetic parameters were calculated by the noncompartmental method using Phoenix™ WinNonlin^®^ Build 6.4 (Certara L.P., St. Louis, MO) software. The actual time of sampling was used in the calculations. The zero time was the start of the first inhalation of the study treatment.

Total systemic exposure of salmeterol and fluticasone propionate was assessed as surrogate for safety by calculating the maximum concentration of concentration–time curve (C_max_) and area under the concentration–time curve from time zero to the last sample with quantifiable concentration (AUC_t_) as the primary variables. For fluticasone propionate, the same parameters determined both the total systemic exposure and lung deposition (surrogate for efficacy) due to its negligible gastrointestinal (GI) bioavailability. For salmeterol, lung deposition was assessed using area under the concentration–time curve from time 0 to 30 minutes after study treatment administration (AUC_30min_) and C_max_ as the primary variables. Charcoal was not used to block GI absorption.

For both active substances, the secondary pharmacokinetic parameter was the area under the concentration–time curve from time zero to infinity (AUC_∞_) determined by adding AUC_t_ to the extrapolated area that was calculated dividing the last quantifiable concentration by the terminal elimination rate constant from log-linear portion of a concentration–time curve (λ_z_.). The other secondary parameters were the time to reach the maximum concentration (t_max_) and the terminal elimination half-life (t_1/2_) calculated using the equation ln2/λ_z_.

Clinical safety was assessed by supine heart rate, systolic and diastolic blood pressure, 12-lead electrocardiogram (ECG), physical examination, and laboratory safety assessments during the screening and end-of-study visits. Adverse events (AEs) were monitored during the whole study. Safety laboratory measurements were analyzed at the study center and United Medix Laboratories Ltd. (Espoo, Finland).

### Statistical methods

Sample size calculation was based on previous studies on the developmental Salmeterol/fluticasone Easyhaler formulations. The assumptions used were significance level 0.0333 (due to multiplicity correction), power 90%, coefficient of variation (CV%) 30, 5% difference between test and reference products, and dropout rate 5%. With these assumptions, a sample size of 64 was selected.

Per-protocol (PP) population was used when comparing the pharmacokinetic parameters.

All the statistical tests were performed in a confirmatory manner according to preplanned statistical hypotheses. Each test product was tested against the reference product. Hochberg multiplicity correction was used to adjust significance level.^(11)^

With this method, the significance level was adjusted sequentially according to the number of tests applied. According to Hochberg, where there are three comparisons, the first confidence level is (1 − α), where α is the chosen significance level. If the null hypothesis cannot be rejected for all the products, the second confidence level is (1 − α/2) for two remaining null hypotheses and the third confidence level is (1 − α/3) for the last hypothesis test. This method was applied according to Zheng et al.^(12)^ to the BE study wherein all the BE comparisons are based on two one-sided tests (TOSTs) and all the noninferiority comparisons are based on one-sided tests.

The primary pharmacokinetic parameters (salmeterol AUC_t_, AUC_30min_, and C_max_ and fluticasone propionate AUC_t_ and C_max_) were analyzed after a logarithmic transformation. An analysis of variance model was fitted for the primary pharmacokinetic parameters.

For noninferiority, a one-sided 95% confidence interval (CI) (i.e., the upper interval for 90% CI) was obtained from the model, and the lower interval for 90% CI is also reported. For BE, two-sided 90% CI for the treatment difference (test–reference) was as well obtained from the model. The confidence level was adjusted according to Hochberg's method, that is, at the first step for BE, two-sided α = 0.1 (noninferiority one-sided α = 0.05); at the second step for BE, two-sided α = 0.05 (noninferiority one-sided α = 0.025); and at the third step for BE, two-sided α = 0.0033 (noninferiority one-sided α = 0.0167).

Noninferiority was accepted if the resulting upper interval of CI laid completely <1.25 and BE was accepted and if CI laid completely between 0.80 and 1.25. If noninferiority for salmeterol C_max_ and AUC_t_ and BE for fluticasone propionate C_max_ and AUC_t_ were shown for the test product when compared with the reference product, BE hypothesis was applied for salmeterol C_max_ and AUC_30min_. The secondary pharmacokinetic variable AUC_∞_ was analyzed analogously to primary variables. Descriptive statistics are presented for t_max_ and t_1/2_.

## Results

### Subject demographics

A summary of demographic and baseline characteristics is presented in Table 1. A total of 73 subjects were screened for the study, with 65 subjects (32 males and 33 females) were subsequently enrolled. All subjects were Caucasian and the mean age was 24.3 years (range 18–48 years). Sixty-one subjects completed the study. Four subjects discontinued the study, one subject due to an AE (mononucleosis infection) and three for other reasons. The safety population included all the 65 randomized subjects. The modified intent-to-treat population included 64 subjects as 1 subject discontinued during period 1 right after dosing and did not provide any relevant measurements. All protocol deviations were minor and did not lead to exclusion from the PP analyses.

**Table 1.** Summary of Demographic Data (Safety Data Set)

	*Female (*N* = 33)*	*Male (*N* = 32)*	*Total (*N = *65)*
Caucasians (*n*)	33	32	65
Mean age (range) years	23.1 (18–29)	25.6 (20–48)	24.3 (18–48)
Mean weight (range) (kg)	66.4 (52–85)	79.2 (66–100)	72.7 (52–100)
Mean height (range) (cm)	167.9 (156–178)	180.1 (171–191)	173.9 (156–191)
Mean body mass index (range) (kg/m^2^)	23.50 (19.7–29.0)	24.38 (20.6–28.7)	23.94 (19.7–29.0)
Mean FEV_1_ of predicted value (range) (%)	92.88 (82.0–108.0)	96.19 (82.0–116.0)	94.51 (82.0–116.0)

FEV_1_, forced expiratory volume in 1 second.

### Pharmacokinetic results

All study treatment administrations were deemed successful and there was no need to reschedule any treatment period.

**FIG. 1.**
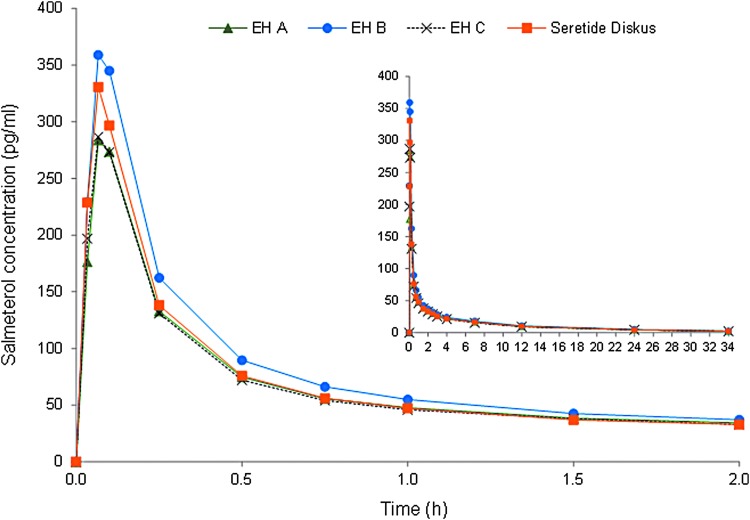
Mean salmeterol plasma concentrations after a single dose administration of 2 × 50/500 μg. Time (hours) in the *x*-axis = 0–2 hours (t = 0–34 hours in a small figure). EH A, EH B, and EH C = Salmeterol/fluticasone Easyhaler products A, B, and C, Reference = Seretide Diskus (*N* = 59).

**FIG. 2.**
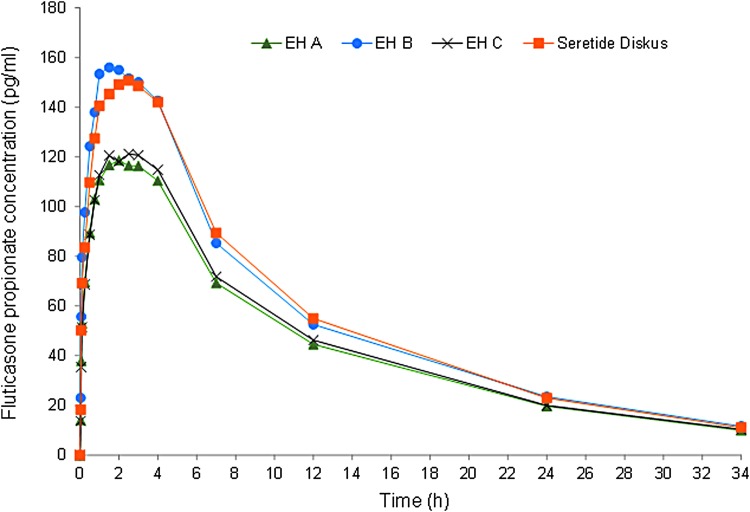
Mean fluticasone propionate plasma concentrations after a single dose administration of 2 × 50/500 μg. Time (hours) in the *x*-axis = 0–34 hours. EH A, EH B, and EH C = Salmeterol/fluticasone Easyhaler products A, B, and C, Reference = Seretide Diskus (*N* = 61).

The concentration–time curves after study treatment administrations for salmeterol and fluticasone propionate are shown in Figures 1 and 2, respectively. The mean concentrations of salmeterol and fluticasone propionate were on a lower level after administration of Easyhaler products A and C than after administration of Easyhaler product B and the reference product.

Salmeterol concentration peak appeared shortly after administration, the median t_max_ being 4.0 minutes for all the study treatments (Table 2). The rate of salmeterol elimination from plasma was similar after all the study treatments (t_½_ was 11 hours). The fluticasone propionate concentration peaked later than salmeterol and the median t_max_ was 1.5 hours for Easyhaler product B and 2.0 hours for the other study treatments (difference not statistically significant). The fluticasone propionate t_½_ values varied between 9 and 10 hours.

**Table 2.** Summary Results of t_max_ and t_1/2_ of Salmeterol and Fluticasone Propionate

	*Salmeterol (*N* = 59)*		*Fluticasone propionate (*N* = 61)*	
	*t_max_ (minutes) Median (range)*	*t_1/2_ (hours) Mean* ± *SD*	*t_max_ (hours) Median (range)*	*t_1/2_ (hours) Mean* ± *SD*
Seretide Diskus	4.00 (2.00–6.00)	10.72 ± 2.04	2.00 (0.1–4.0)	9.33 ± 1.71
Easyhaler A	4.00 (2.00–15.00)	10.77 ± 1.96	2.00 (0.3–4.0)	9.95 ± 1.42
Easyhaler B	4.00 (4.00–6.00)	11.00 ± 2.44	1.50 (0.1–4.0)	10.03 ± 1.61
Easyhaler C	4.00 (2.00–6.00)	10.63 ± 2.57	2.00 (0.3–4.0)	9.77 ± 1.56

A single dose of 2 × 50/500 μg/inhalation was administered.

t_max_, the time to maximum concentration; t_1/2_, the terminal elimination half-life; SD, standard deviation.

Statistical analyses and comparisons between the test and the reference products were carried out first for the total systemic exposure of salmeterol. The PP population comprised 59 subjects as 4 subjects discontinued prematurely and 2 subjects had insufficient number of pharmacokinetic samples at the time of peak concentration, not allowing reliable estimate of salmeterol peak exposure. The upper interval for 90% CI for the test/reference ratios of salmeterol C_max_, AUC_t_, and AUC_∞_ was <1.25 for all the comparisons (Table 3). Hence, all the test products can be declared to be noninferior compared with the reference product in salmeterol total systemic exposure.

**Table 3.** Summary Results of Salmeterol C_max_, AUC_t_, and AUC_∞_ Comparisons (Estimated Geometric Means, *N* = 59)

	*C_max_*	*T/R*	*90% CI*^a^	*AUC_t_*	*T/R*	*90% CI*^a^	*AUC_∞_*^b^	*T/R*	*90% CI*^a^
Seretide Diskus	319			426			464		
Easyhaler A	282	0.8834	0.8286–0.9419	415	0.9752	0.9305–1.0221	451	0.9725	0.9291–1.0178
Easyhaler B	359	1.1266	1.0567–1.2012	481	1.1286	1.0769–1.1829	525	1.1324	1.0819–1.1852
Easyhaler C	282	0.8836	0.8287–0.9420	408	0.9590	0.9150– 1.0051	445	0.9596	0.9169–1.0044

A single dose of 2 × 50/500 μg/inhalation was administered.

^a^ The noninferiority acceptance limit for the upper level for 90% CI was 1.25.

^b^ The percentage of AUC_∞_ observations with >20% extrapolated was 0.8.

C_max_, the maximum concentration of concentration–time curve (pg/mL); AUC_t_, area under the concentration–time curve from time zero to the last sample with quantifiable concentration (pg·h/mL); AUC_∞_, the area under the concentration–time curve from time zero to infinity (pg·h/mL); T/R, test/reference ratio; CI, confidence interval.

For fluticasone propionate, PP population included 61 subjects as 4 subjects discontinued prematurely. The statistical analyses of the primary pharmacokinetic parameters of fluticasone propionate C_max_ and AUC_t_, and the secondary parameter AUC_∞_ for lung deposition and systemic exposure comparisons are shown in Table 4.

**Table 4.** Summary Results of Fluticasone Propionate C_max_, AUC_t_ and AUC_∞_ Comparisons (Estimated Geometric Means, *N* = 61)

	*CI (%)*^a^	*C_max_*	*T/R*	*CI*^b^	*AUC_t_*	*T/R*	*CI*^b^	*AUC_∞_*^c^	*T/R*	*CI*^a^
Seretide Diskus		158			1835			1985		
Easyhaler A	90.0	126	0.7952	0.7548–0.8377	1469	0.8005	0.7675–0.8350	1607	0.8096	0.7764–0.8442
Easyhaler B	90.0	168	1.0594	1.0057–1.1161	1831	0.9980	0.9568–1.0410	2009	1.0120	0.9705–1.0553
Easyhaler C	90.0	130	0.8225	0.7807–0.8665	1515	0.8258	0.7917–0.8614	1656	0.8344	0.8001–0.8701
Seretide Diskus		158			1835			1985		
Easyhaler B	95.0	168	1.0594	0.9956–1.1274	1831	0.9980	0.9491–1.0495	2009	1.0120	0.9627–1.0639
Easyhaler C	95.0	130	0.8225	0.7729–0.8752	1515	0.8258	0.7853–0.8684	1656	0.8344	0.7937–0.8771
Seretide Diskus		158			1835			1985		
Easyhaler B	96.7	168	1.0594	0.9901–1.1336	1831	0.9980	0.9448–1.0542	2009	1.0120	0.9584–1.0686

A single dose of 2 × 50/500 μg/inhalation was administered.

^a^ Confidence level according to multiplicity correction method.

^b^ The BE (bioequivalence) acceptance range for the CIs was 0.80–1.25.

^c^ The percentage of AUC_∞_ observations with >20% extrapolated was 1.2%.

C_max_, the maximum concentration of concentration–time curve (pg/mL); AUC_t_, area under the concentration–time curve from time zero to the last sample with quantifiable concentration (pg·h/mL); AUC_∞_, the area under the concentration–time curve from time zero to infinity (pg·h/mL); T/R, test/reference ratio; CI, confidence interval.

When the test products were compared with the reference product using an α value of 0.10 (90% CI), only Easyhaler test product B met the BE criteria (90% CI 0.80–1.25). For the second testing step, Easyhaler test product A, which differed most from the reference, was excluded and an α value of 0.05 was used (95% CI) in test/reference comparisons. In the second testing step, only Easyhaler test product B was bioequivalent with the reference product. In the last phase of the analysis, the test product differing more from the reference product, Easyhaler test product C, was excluded and the remaining test product, Easyhaler product B, was tested against the reference product with the α value of 0.033. Easyhaler product B was bioequivalent with the reference product in efficacy and safety of fluticasone propionate with 96.7% confidence level.

According to the statistical plan, test/reference comparisons for salmeterol efficacy (lung deposition) were performed for the test products fulfilling the criteria for both preceding analyses, noninferiority in salmeterol systemic exposure, and BE in fluticasone propionate lung deposition and systemic exposure. The Easyhaler product B/reference comparisons for the estimated geometric means of salmeterol C_max_ and AUC_30min_ are shown in Table 5. Easyhaler product B met the BE criteria for salmeterol lung deposition in comparison with the reference product. Therefore, Easyhaler product B fulfilled all the preset noninferiority and BE criteria.

**Table 5.** Summary Results of Salmeterol C_max_ and AUC_30min_ Comparisons (Estimated Geometric Means, *N* = 59)

	*C_max_*	*T/R*	*90% CI*^a^	*AUC_30min_*	*T/R*	*90% CI*^a^
Seretide Diskus	319			79		
Easyhaler B	359	1.1266	1.0567–1.2012	92	1.1605	1.0989–1.2255

A single dose of 2 × 50/500 μg/inhalation was administered.

C_max_, the maximum concentration of concentration–time curve (pg/mL); AUC_30min_, area under the concentration–time curve from time 0 to 30 minutes after administration (pg·h/mL); T/R, test/reference ratio; CI, confidence interval.

^a^ The BE (bioequivalence) acceptance range for the 90% CIs was 0.80–1.25.

### Safety

The safety profiles of the test and reference products were similar with regard to AEs and the safety assessments. There was one AE (mononucleosis infection) leading to discontinuation of the study treatment. Altogether, 134 AEs were reported during the study treatment, the most common being headache (61 events), followed by nausea (15 events) and nasopharyngitis (13 events). Six AEs were considered to be related to the study treatment (headache four events, nausea and dizziness one event each). The events were evenly distributed across the treatments. There were no major changes in vital signs, laboratory parameters, and ECG between screening and end of study.

## Discussion

In this study, three different Salmeterol/fluticasone Easyhaler test products were compared against the reference product Seretide Diskus. The aim was to demonstrate that total systemic exposure of salmeterol and fluticasone propionate is not higher after administration of a test product than after the reference product and that the test and the reference product have equivalent lung deposition.

Salmeterol and fluticasone propionate concentrations were similar after administration of the test product B and the reference, and the criteria for the BE were met. The concentrations were significantly lower after two other test products. Median salmeterol t_max_ was at 4 minutes measured from the start of the first inhalation, showing rapid absorption. Fluticasone propionate t_max_ was at 1.5 hours for the test product B, being slightly earlier than for the other products (2.0 hours). The difference between Easyhaler B and the reference is not statistically nor clinically significant. There were no safety concerns during the study.

For the study, three different Salmeterol/fluticasone Easyhaler test products were developed based on the information received from developmental Easyhaler formulations used in the preceding studies. The products had differences in particle size distribution and, in addition, the inhaler of test product B had a slightly different mouthpiece compared with the inhaler of products A and C. The difference was not visible to the subjects and did not change the use of the inhaler but affected the drug particle deagglomeration from the lactose carrier.

It is common practice in the development of a second entry inhaled product to conduct one or several pilot scale studies before entering to a pivotal study. This approach has been proven to be quite successful. However, conducting several studies in a row takes time and pilot studies may not always find the best possible test product for the pivotal study. Applying the multiplicity correction method (here Hochberg multiplicity correction method) provides another option for testing several test products and may save significant amount of development time. Multiplicity adjustment procedure in combination with TOSTs provides a way to design efficient BE crossover trials while type I error is still properly controlled.^(12)^

The study was carried out with a single dose, open, and randomized crossover design. This design has been accepted for pharmacokinetic testing of OIPs by the European authorities.^(13–15)^ The dose was 100 μg of salmeterol and 1000 μg of fluticasone propionate, consisting of two inhalations of 50/500 μg/inhalation strength. Such a dose enabled the determination of plasma drug concentrations up to 34 hours after administration, as the drug concentrations in plasma were sufficiently high. As a result, all the pharmacokinetic parameters, including the elimination half-life, could be assessed reliably.

An open study was justified because otherwise it would not have been feasible to take a pharmacokinetic blood sample as early as 2 minutes after the start of first study treatment inhalation. Frequent sampling right after dosing is essential, since salmeterol concentration peaks very early and at least one sample before maximum concentration is preferred.^(16)^ Open study did not endanger the reliability of the data since the bioanalytical laboratory carrying out the analysis was kept blinded.

The subjects of the study were healthy male and female volunteers. OIP guideline^(6)^ suggests conducting pharmacokinetic studies in the intended patient population. However, this study was carried out in healthy volunteers as the studies found in the literature suggest that BE testing of OIPs in healthy volunteers would be more sensitive than that in COPD or asthmatic patients because pharmacokinetic parameter values are higher in healthy volunteers^(17–20)^ and variability, which is not related to differences between the products, is lower.^(18)^ As a consequence, studies with healthy volunteers allow the demonstration of equivalence with smaller number of subjects and lesser exposure to an investigational medicinal product. Based on the recent approvals of OIPs, this is also the current view of European authorities^(15,21)^ and will be taken into account in the coming revision of the OIP guideline.^(22)^

To assess pulmonary deposition after inhaled administration, absorption of the active substance from the GI tract must often be blocked with charcoal, whereas for total systemic exposure, absorption from both lung and GI tract must be taken into account. However, fluticasone propionate has an oral availability of <1%^(23)^and, therefore, the amount of fluticasone propionate swallowed after inhalation contributes minimally to systemic exposure. Negligible exposure through GI tract has also been confirmed in our earlier studies (data on file). Hence, systemic absorption of inhaled fluticasone propionate occurs mainly through the lungs and administration of charcoal for lung deposition comparisons is not needed.

Charcoal blockade was considered unnecessary in assessing salmeterol lung deposition too. This is because for drugs like salmeterol for which the absorption of the drug in the lung is very quick (t_max_ ≤5 minutes) and occurs before the contribution of GI absorption is significant, AUC_30min_ after administration without charcoal can be used as a measure of lung deposition.^(24)^ In our previous studies with Seretide Diskus salmeterol, t_max_ has occurred in <5 minutes, and thus, administration with charcoal blockade was omitted and a new primary parameter, AUC_30min_, was introduced.

The study is an important element in the development of a new product to the Easyhaler product range. Inhaled corticosteroid/long-acting β_2_-agonist combinations are the main treatment option in asthma from treatment step 3 onward,^(25)^ whereas in patients with moderate to very severe COPD, they are considered appropriate step-up therapy for patients experiencing exacerbations while taking long-acting bronchodilators.^(26)^

Asthma and COPD patients need product options to manage their disease with an inhaler most suitable for them. It has been shown that COPD patients who use inhalers requiring similar inhalation technique have lower rate of exacerbations and are less likely to use higher doses of short acting bronchodilators than those who use inhalers with mixed inhalation techniques.^(27)^

Because of the wide range of products available in the Easyhaler inhaler, it is convenient to switch from one product to another without the need to learn another inhaler technique in case medication adjustments are needed. Inhaler technique is, indeed, an important element of effective drug delivery. That is recognized also in the current treatment guidelines for asthma and COPD, which consider training and continuous assessment of technique as an essential task of healthcare providers when reviewing the patients.^(25,26)^

## Conclusions

In conclusion, Salmeterol/fluticasone Easyhaler (product B) was shown to be bioequivalent with the reference product Seretide Diskus. The safety of all test products was good and comparable with the reference product.

## Notes

Reviewed by:

Bo Olsson

Peter Daley-Yates
